# Dynamic behavior of reversible oxygen migration in irradiated-annealed high temperature superconducting wires

**DOI:** 10.1038/s41598-020-70663-1

**Published:** 2020-09-10

**Authors:** Yi Zhang, M. W. Rupich, Vyacheslav Solovyov, Qiang Li, Amit Goyal

**Affiliations:** 1 Research and Education in Energy, Environment and Water (RENEW), State University of New York (SUNY), Buffalo, NY 14260 USA; 2 AMSC, 114 E. Main St. Ayer, Massachusetts, 01432 USA; 3 Brookhaven Technology Group, 1000 Innovation Rd., Stony Brook, NY 11794 USA; 4 Condensed Matter Physics and Materials Sciences Department, Brookhaven National Laboratory, Upton, NY 11973 USA; 5 Department of Chemical and Biological Engineering, State University of New York (SUNY) at Buffalo, Buffalo, NY 14260 USA

**Keywords:** Engineering, Materials science, Nanoscience and technology

## Abstract

We use atomically resolved scanning transmission electron microscopy and electron energy loss spectroscopy to determine the atomic-scale structural, chemical and electronic properties of artificial engineered defects in irradiated-annealed high temperature superconducting wires based on epitaxial Y(Dy)BCO film. We directly probe the oxygen vacancy defects in both plane and chain sites after irradiation with 18-meV Au ions. The plane site vacancies are reoccupied during post-annealing treatment. Our results demonstrate the dynamic reversible behavior of oxygen point defects, which explains the depression and recovery of self-field critical current and critical temperature in irradiation-annealing process. These findings reveal the strong effect of oxygen vacancies in different sites on the superconductivity properties of irradiated Y(Dy)BCO film, and provide important insights into defects engineering of 2G HTS coil wires.

## Introduction

Since the discovery of high temperature superconducting (HTS) rare earth barium copper oxide REBa_2_Cu_3_O_7-δ_ (REBCO), significant efforts have been made to enhance its functionalities by introducing artificial vortex pinning microstructures. A variety of defects in the superconducting films have been proven to serve as effective pinning centers^1^. For example, inhomogeneous strained regions associated with intergrowths induce Cooper pair suppression, and vortex-pinning centers are generated in these regions^2^. Two major approaches have been developed in past decades to optimize vortex pinning landscape by producing highly engineered defects structures. One approach is to synthesize HTS composites containing self-assembled non-superconducting oxide nanorods within the superconductor matrix^3–6^. For instance, by using YBa_2_Cu_3_O_7-δ_ (YBCO) source material containing excess Ba and Zr, the metal oxides BaZrO_3_ (BZO) will be deposited simultaneously with YBCO. Improvement of vortex-pinning via the incorporation of non-superconducting, self-assembled nanocolumns is accomplished by a phase-separation and strain-driven self-assembly process. The ordered array of self-assembled nanorods is formed under compressive strain due to lattice mismatch between the embedded phase and surrounding matrix. The resulting BZO nanocolumns extend along c-axis with a width approximately two times of the superconducting coherence length ξ. These nanocolumns give rise to perfect correlated pinning centers for the composite films and produce a significant c-axis-correlated enhancement of critical current density *J*_*c*_.

An alternative approach is to introduce defects structures using electron, proton, neutron, or ion irradiation^7–11^. The irradiation particles collide with YBCO to displace the surrounding atoms and generate defects. Depending on the mass and energy of particles, the irradiation can introduce various type of artificial irradiation defects. Hundreds of MeV to GeV heavy ions are required to generate columnar defects, whereas the lower energy ions, electrons, and protons can introduce random point defects and nano-scale defects^12^. The effects of different irradiation defects on vortex pinning in epitaxial films have been widely studied^13^. Introducing artificial irradiation defects can achieve significant enhancement in *J*_*c*_, with a cost of depression of critical temperature *T*_*c*_. The effects are related to the degradation of film quality caused by the defects. By optimizing the parameters of chemical synthesis and particle irradiation, the depression can be controlled. On the other hand, annealing processes can lead to a recovery of *T*_*c*_^14–16^. The amorphous columnar defects remain stable, whereas oxygen disorders and point defects, restored by post-annealing, contribute to the improvement of film quality and superconductivity properties. Although irradiation defects have been studied over a full range of atomic, electronic structures and physical properties^17–21^, a detailed TEM analysis on the annealing effects is still desirable, especially on the commercial HTS coil wires.

**Figure 1:**
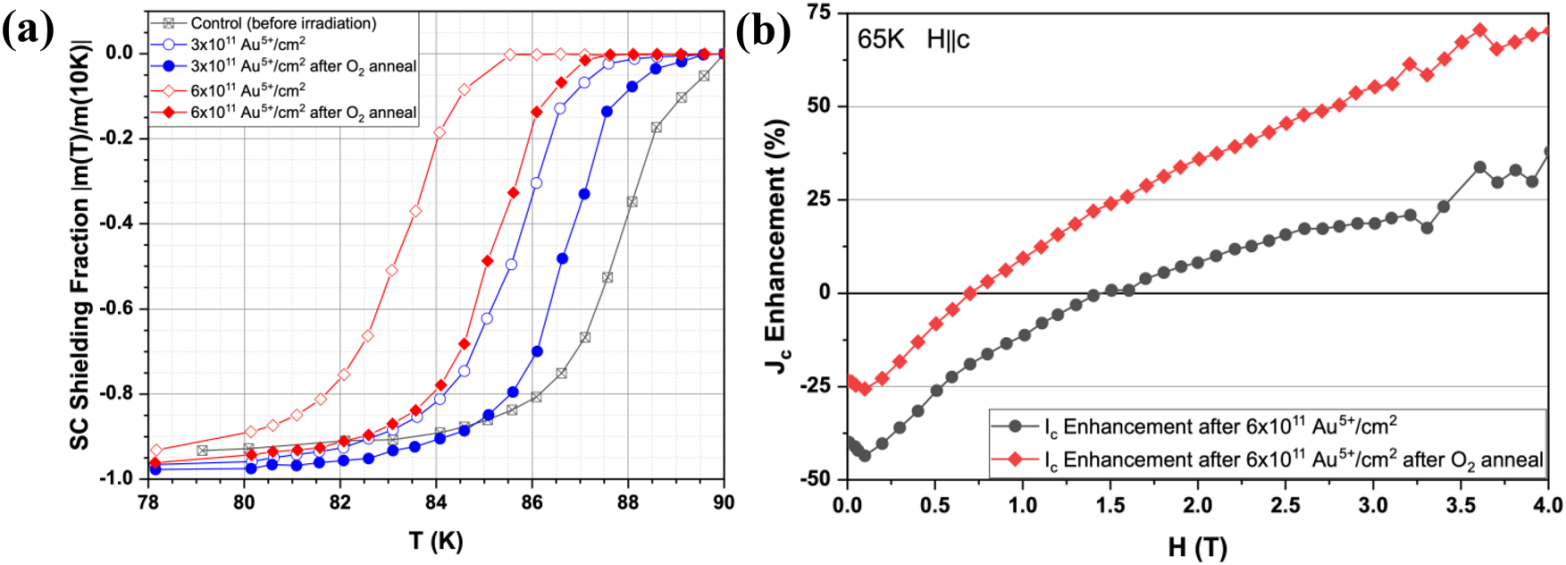
**(a)** Temperature dependence of the superconducting shielding fraction in YBCO films. **(b)** The *J*_*c*_ enhancement versus applied magnetic field at 65 K for the irradiated and annealed samples.

In this work, we present a detailed investigation of the post-annealing effect on irradiation defects in AMSC’s second generation (2G) HTS coil wires. 1.2 µm thick Dy-doped YBCO films were deposited by a metal organic deposition (MOD) process onto a 46 mm wide oxide buffered Ni-5at%W RABiTS substrate produced by Rolling-Assisted-Biaxially-Textured Substrate (RABiTS) process as described elsewhere^22–24^. The HTS strip was irradiated in a reel-to-reel process at Brookhaven National Laboratory using the tandem Van de Graaff accelerators with 18 meV Au^5+^ ions to a dosage of 3 × 10^11^ Au^5+^/cm^2^ along the c-axis of the Y(Dy)BCO film^22^. According to The Stopping and Range of Ions in Matter (SRIM-2008) simulation results, the Bragg peak is located inside the Y(Dy)BCO film, while radiation induced defects should be quite uniform (variation ~ 30%) throughout Y(Dy)BCO thickness. The irradiation results in a decrease in T_c_, which increases with the dosage, as seen in Fig. 1a. The 77 K, self-field *I*_*c*_ of the sample also decreases after irradiation, falling from around 385 A/cm-w in the original sample to about 320 A/cm-w after irradiation with a dose of 3 × 10^11^ Au^5+^/cm^2^. The irradiated samples were then annealed in oxygen atmosphere at 170 ℃ for 60 min, which led to an increase in *T*_*c*_ (Fig. 1a) and an enhancement in 77 K, self-field *I*_*c*_ to around 358 A/cm-w. The oxygen annealing process also enhances the *I*_*c*_ of the samples in the presence of a magnet field as seen in Fig. 1b. The annealing process provides an additional low-cost way to improve *I*_*c*_ which could be incorporated into the roll-to-roll manufacturing. In this paper, atomic and chemistry structures of irradiation defects are studied by employing aberration-corrected scanning transmission electron microscopy (AC-STEM) coupled with atomic resolution electron energy loss spectroscopy (EELS). We demonstrate the reversible oxygen migration during radiation-annealing processes, which leads to a significant change in the superconducting properties between as-irradiated sample and annealed-irradiated sample. The TEM work clearly reveals how the low energy heavy ion irradiation and post-annealing process can result in a remarkable improvement of the critical current of AMSC’s standard 2G coil wire.

## Results and discussion

### Morphologies of preexisting defects

**Figure 2:**
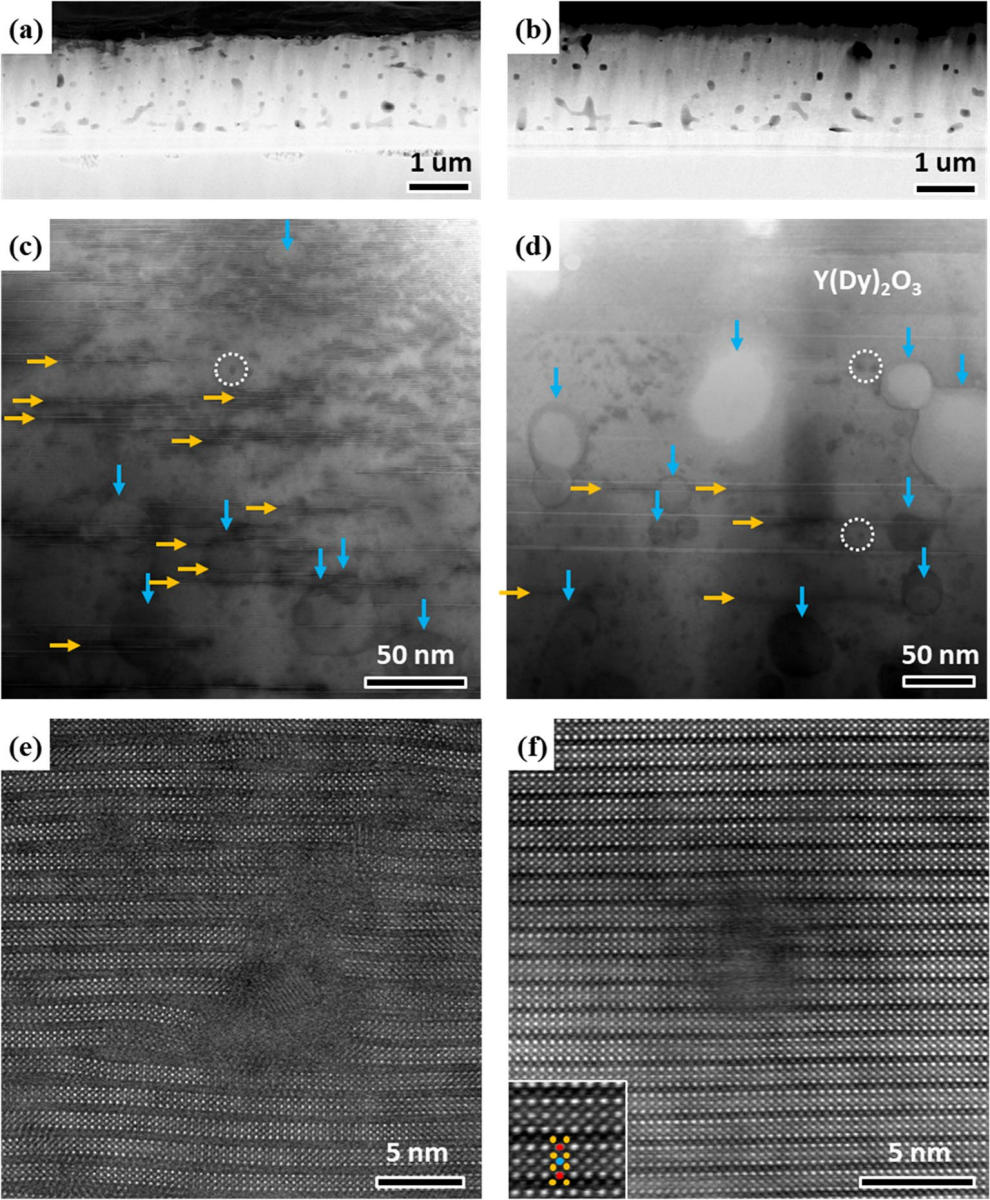
Low magnification STEM images of **(a)**, **(c)** as-irradiated and **(b)**, **(d)** annealed Y(Dy)BCO. High density of voids, Y(Dy)_2_O_3_ nanoparticles, (vertical arrows), planar defects (horizontal arrows) and irradiation-induced clusters (dotted circles) are observed in both films. **(e)** and **(f)** are atomic-resolution HAADF images of irradiated amorphous defects in as-irradiated and annealed film, respectively. The bottom left inset of **(f)** indicates the unit cell of Y(Dy)BCO.

Figure 2 shows the morphology of as-irradiated and annealed samples. As expected, there is no difference between the morphologies of two samples at the microscale level. The MOD processing incorporates a dispersion of voids, nanoparticles, stacking faults and planar defects into Y(Dy)BCO wires^25^. These pre-existing defects are observed in both films. The films contain Y(Dy)_2_O_3_ nanoparticles with the size range of 10–40 nm, which provide strong pinning in the as-grown films. A high density of stacking faults, identified as Y_1_Ba_2_Cu_4_O_8_ (Y124) intergrowths, with extra Cu–O chains are seen in higher resolution HAADF images (Figure S1). The stacking faults diameters are found in the range of 5-20 nm. This high density of stacking faults has been reported to be the cause of an extended local structure distortion area where vortices are effectively pinned^2^. The distortion can extend up to ~ 2 nm, corresponding to 2 unit cells, from the intergrowths. In coil wires, the stacking faults are separated by ~ 1–2 unit cells along c-axis. This demonstrates that the pre-irradiation films are highly strained and pinning enhanced results from the preexisting defects.

A dispersion of irradiation-induced nanoclusters is present in the as-irradiated and annealed films. In contrast, these features are not observed in the non-irradiated film (Figure S2). We have collected more than 50 images from different areas of the non-irradiated film to confirm this conclusion. This reveals that the annealing temperature is not high enough to annihilate the nanoclusters, which requires cation-diffusion. As previous studies reported, the annealing condition would predominantly lead to a reduction of point defect density, which is invisible to TEM^26,27^. Atomic resolution STEM images illustrate the morphology of irradiation defects as shown in Fig. 2e, f. The defects in the irradiated Y(Dy)BCO film are amorphous nanoclusters with a size of around 2–5 nm. The amorphous nanoclusters are surrounded by highly distorted YBCO layers, which could be caused by stress relaxation. In comparison, the YBCO around Y(Dy)_2_O_3_ nanoparticles did not show such features. From the TEM results, we estimated a density of the amorphous nanocluster to be between 1.1 × 10^11^ to 3.6 × 10^11^/cm^2^ with an average size of 2.14 nm and a spacing of about 50 nm. The irradiation defects in annealed film have the same size range as the defects in as-irradiated film. Most of the irradiation defects are amorphous, while some of them become partially reoxygenated after annealing.

### Annealing effect on defects structure and composition

**Figure 3:**
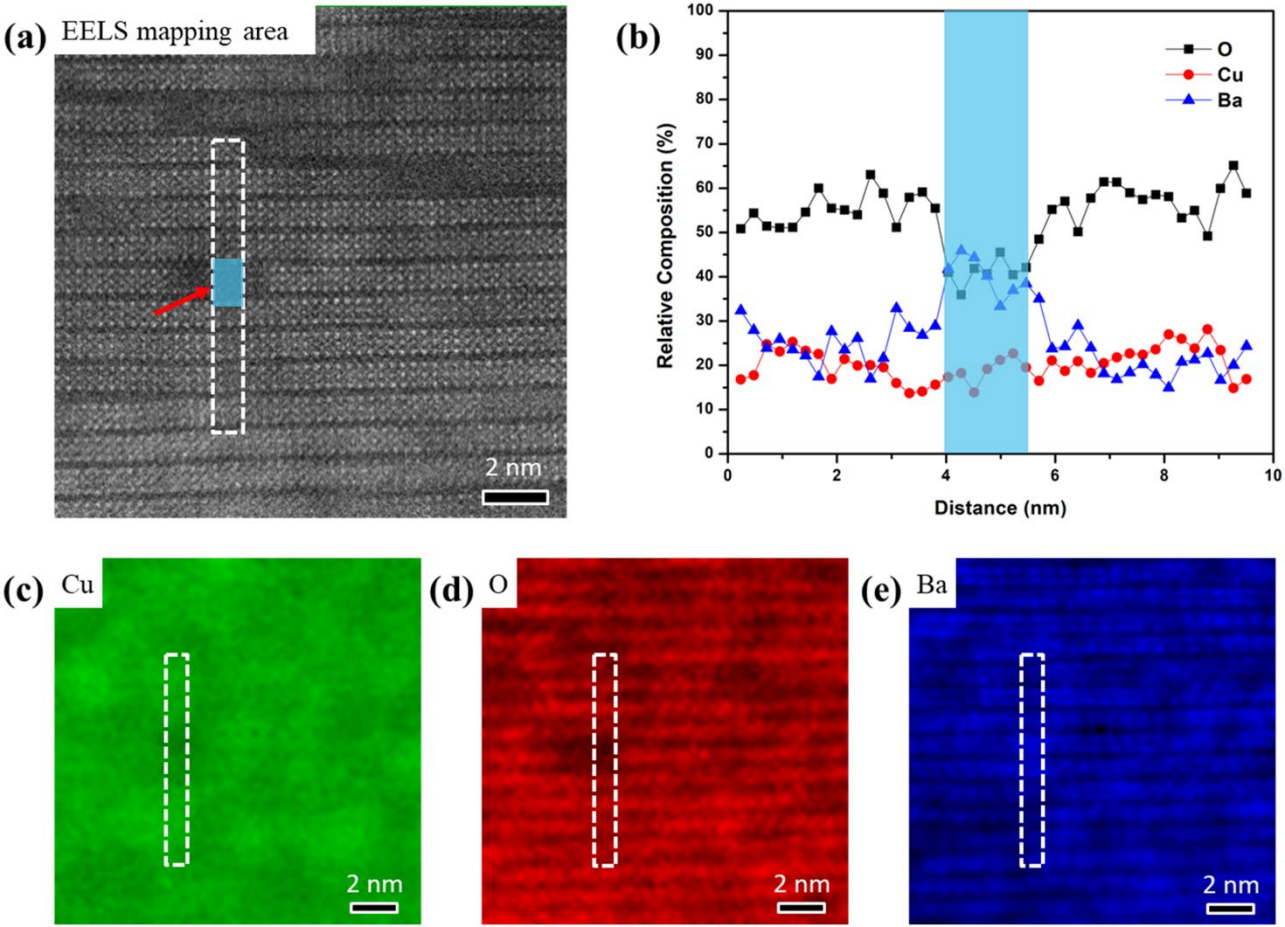
**(a)** A 2-D EELS map acquired from the HAADF STEM image includes both defect (indicated by arrow) and surrounding YBCO matrix in irradiated film. **(b)** HAADF intensity profile and Cu, O, and Ba are summed over the boxed region shown in **(a)**. Relative composition maps of Cu-*L*_*2, 3*_, O-*K*, and Ba-*M*_*4, 5*_ are shown in **(c)**–**(e)**, respectively.

To investigate annealing effect on the irradiation-induced defects, the spatial variations of EELS spectra in both as-irradiated and annealed samples are acquired. The EELS map that includes both defects and surrounding YBCO matrix is analyzed (Fig. 3a) with a spatial step size of 0.24 nm. Two-dimensional relative composition maps of Ba-*M*_*4, 5*_, Cu-*L*_*2, 3*_, and O-*K* edges are shown in Fig. 3c–e, respectively. We noticed that the intensity of O-*K* map decreased at the defect regions, indicating a decrease of the O concentration. For clarity, the relative composition of Cu, O, and Ba across the defect are extracted from the dash line box area, which includes ~ 2 × 9 unit cells. An intensity profile is summed over the same area in the HAADF image. The quantitative EELS analysis determined from the ratios of integrated intensities of Ba, Cu, and O (Fig. 3b) shows a dramatically lower O in the defect region. The calculated Ba:Cu:O ratio of YBCO matrix is ~ 1:1:3, which is close to the stoichiometric proportion within experimental error. For comparison, we also acquired EELS spectra from perfect YBCO matrix in non-irradiated film, as shown in Fig. S3. The elemental distributions before irradiation are homogeneous, which has the same Ba:Cu:O ratio as that of YBCO matrix in irradiated film. In contrast, the irradiation induced defect reveals an oxygen deficient region. The findings confirm that the low energy, heavy ion irradiation damaged the local structure, causing oxygen anion diffusion in the damaged area.

**Figure 4:**
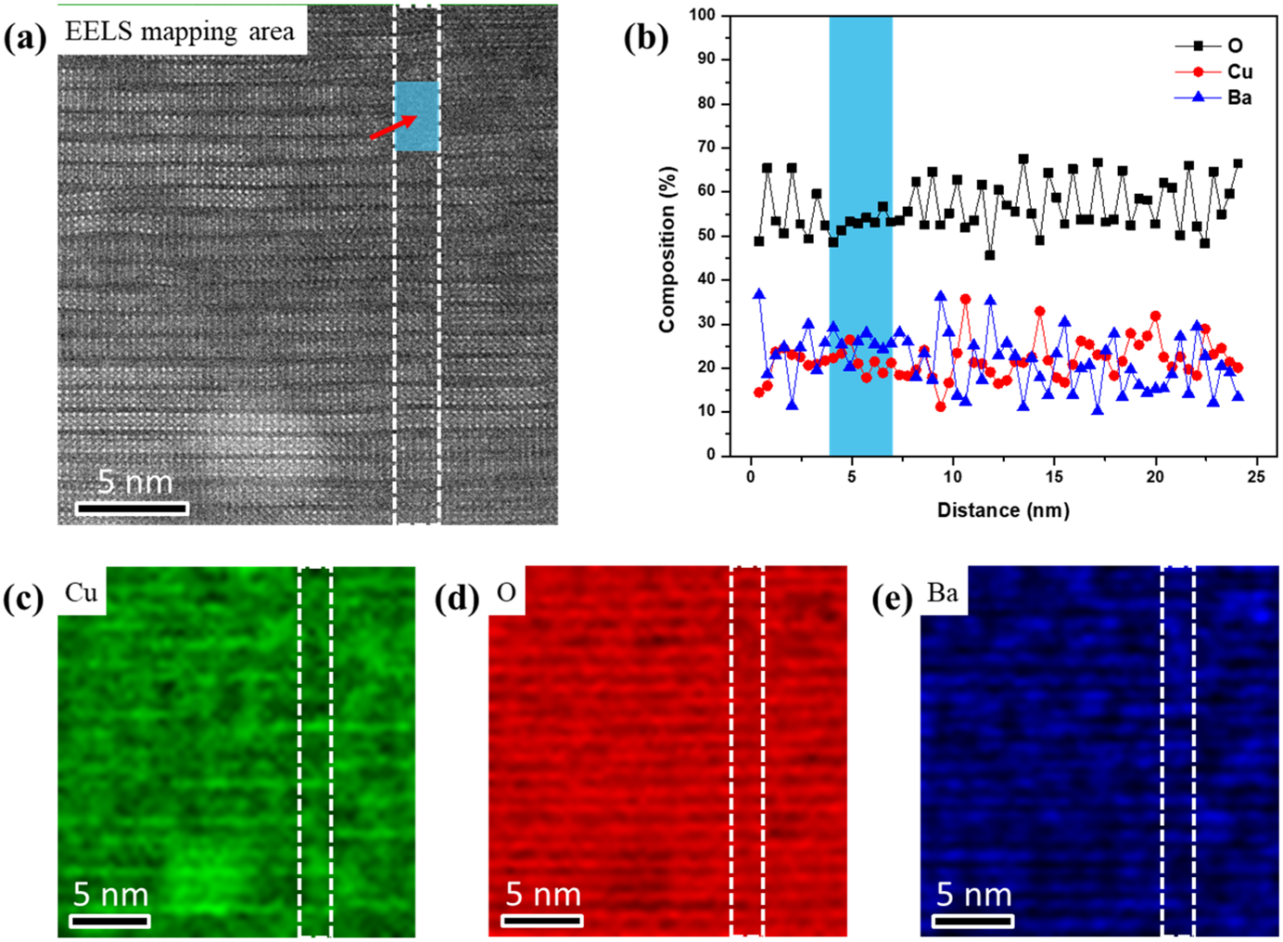
**(a)** Two-dimensional EELS map is collected from annealed sample. The area includes both defect (indicated by arrow) and surrounding YBCO matrix. **(b)** Elements distributions are extracted from the dashed line box by summing the spectra to reduce noise. The relative composition profile does not show obvious change at defect region (colored area), which indicates the recovery of oxygen concentration after annealing in oxygen atmosphere. The relative composition maps of Cu, O, and Ba are shown in **(c)**, **(d)**, and **(e)** respectively.

Two-dimensional EELS map is also acquired from the annealed sample as shown in Fig. 4. Figure 4c–e show the relative composition maps for Ba, Cu, and O, respectively. Compared to the significant intensity variation at defect region in the as-irradiated film, the intensity of O in Fig. 4d does not show any obvious change at the defect region. We have profiled the distribution of Ba, Cu, and O across the defect, as shown in Fig. 4b. The relative composition of Ba, Cu, and O has been extracted from the dashed line box region by summing the spectra to reduce noise. The elements’ relative compositions are nearly unchanged across the defect, which indicates no obvious oxygen deficiency. It’s worth noting that EELS is a highly localized technique that probes the local chemical information. The defect area, where the perfect YBCO structure is damaged, has a smooth relative composition. More EELS data sets collected from both as-irradiated and annealed films are presented in Fig. S4. The observations illustrate the dynamic reversible behavior of oxygen atoms in the irradiated YBCO film. Collisions of Au ions with YBCO matrix lead to the displacement of atoms, mostly O and Cu atoms. The local structure loses its crystallinity and forms nanoscale non-stoichiometric amorphous defects. Subsequent annealing in an oxygen atmosphere at a temperature of 170 ℃ does not fully crystalize the defect regions but is sufficient to restore the oxygen concentration, leaving a stoichiometric disordered lattice.

**Figure 5:**
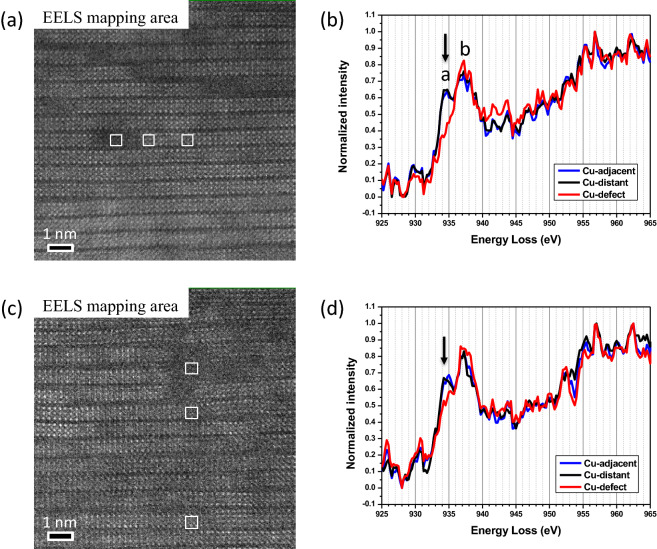
Cu-*L*_*2,3*_ edge fine structures are extracted from the marked defect, adjacent matrix, and distant matrix areas in both **(a)** as-irradiated and **(c)** annealed samples, by summing 3 × 3 pixels (3 unit cells). The spectra are plotted in **(b)** and **(d)**, respectively. In as-irradiated sample **(b)**, the Cu *L*_*3*_ edge spectra from adjacent and distant matrix areas show splitting peaks at 934.5 eV (arrowed peak *a*) and 937.2 eV (peak *b*), resulting from the existence of mixed valence states of Cu^2+^ and Cu^1+^ ions in Y(Dy)BCO matrix. The irradiation defect area, which shows only peak *b*, indicates oxygen deficiency transforming Cu from 2 + to 1 + . In annealed film **(d)**, the spectra show enhanced intensity peak *a*, which manifests that the oxygen doping commonly exists in whole film.

The irradiation induced variation in the stoichiometry and crystalline structure of defects can be understood by studying the detailed electronic structure of the irradiation defects. EELS spectra from defect, adjacent matrix, and distant matrix areas in both as-irradiated and annealed samples are plotted in Fig. 5. The Cu-*L*_*2,3*_ edge fine structures are extracted from spectrum images by summing 3 × 3 pixels (3 unit cells). Cu *L*_*3*_ and *L*_*2*_ white lines are very sensitive to the change in valence states. In the as-irradiated sample, the Cu *L*_*3*_ edge spectra from adjacent and distant matrix areas shows splitting peaks at 934.5 eV (arrowed peak *a*) and 937.2 eV (peak *b*), resulting from the existence of mixed valence states of Cu^2+^ (peak *a*) and Cu^1+^ (peak *b*) ions in Y(Dy)BCO matrix. It is worth noting that only peak *b* is observed in the Cu *L*_*3*_ edge from irradiation defect area where oxygen is highly deficient. The diminished peak *a* indicates that the irradiation induced oxygen deficiency transforms Cu in Y(Dy)BCO structure from Cu^2+^ to Cu^1+^. Compared with the spectrum of irradiation defects in the as-irradiated sample, a conspicuous peak *a* is observed in the Cu *L*_*3*_ edge from the annealed defect area. The presence of Cu^2+^ (peak *a*) is consistent with the recovery of oxygen concentration. Furthermore, the Cu *L*_*3*_ edge spectra from Y(Dy)BCO matrix also show an enhanced intensity around peak *a*, which manifests the oxygen doping that commonly exists in whole film.

### Annealing effect on YBCO electronic structure

**Figure 6:**
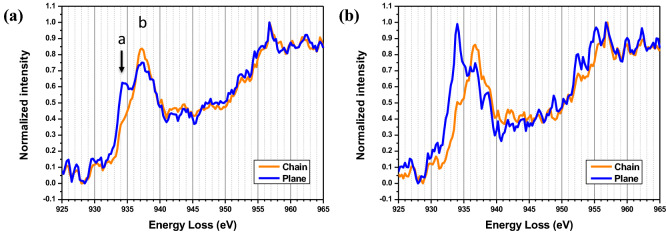
The EELS Cu-*L*_*2,3*_ edge fine structure of chains and planes in irradiated film **(a)** and annealed film **(b)** are extracted from the region away from nanoscale defects. The peaks labeled *a* and *b* indicates the Cu^2+^ and Cu^1+^ oxidation states.

It is important to investigate the oxygen behavior by probing electronic structure of different sites in Y(Dy)BCO matrix. We extracted the Cu-*L*_*2,3*_ edge spectra from different layers of Y(Dy)BCO structure in the irradiated film to clarify the location of different valence states of Cu (Fig. 6a). The Cu spectrum in the chain layer shows a shoulder peak at 934.5 eV (peak *a*) and a main peak at 937.2 eV (peak *b*), which corresponds to predominant Cu^1+^ ions in Cu–O chains. Cu *L*_*3*_ edge in the spectrum of CuO_2_ plane layer shows splitting peaks *a* and *b*, which indicates the coexistence of Cu^2+^ and Cu^1+^ oxidation states. As a comparison, we also collected Cu-*L*_*2,3*_ edge spectra from non-irradiated sample (Figure S5). The spectra show splitting *L*_*3*_ edge in chain layer and peak *a* in plane layer, which is consistent with the previous reported YBCO_7_ data^28^. Pre-irradiation oxygen vacancies, formed during film synthesis process, generally reside at the chain sites. In YBCO_7-δ_ from δ = 1 (full chains) to δ = 0 (empty chains), the oxygen content changes converting the valence of Cu in the chain layers from Cu^2+^ to Cu^1+^, as a result of doping holes into the CuO chain layers. Meanwhile, in the range of δ = 1 to 0, the Cu in CuO_2_ planes does not show a significant change. This can be explained by the effect of different displacement energies for oxygen in different sites. The displacement energy for O in CuO_2_ planes has been evaluated as 8.4 eV, which is about three times that for the chain oxygen (2.8 eV)^29,30^. In this paper, the irradiation species’ energy is above the threshold energy for oxygen displacement, which is supposed to cause point defects in both planes and chains. The oxygen displacement caused by irradiation results in the splitting Cu *L*_*3*_ edge in CuO_2_ plane layers and the substantial reduced peak *a* in CuO chain layers (Fig. 6a). After annealing in oxygen atmosphere, as shown in Fig. 6b, the chain layers still show main peak *b* with a shoulder peak *a*. However, the plane layers have the similar Cu edges as non-irradiated sample. The results indicate the O doping is preferred to re-occupy the O vacancy sites in plane layers.

We investigate the complex pinning landscapes in the irradiated film formed by the pre-existing defects, irradiation-induced clusters and point defects. After annealing, most of the point defects have been restored but the clusters are still present, which leads to the partial recovery of the *T*_*c*_ and self-field *I*_*c*_. Previous reports have done a thorough analysis of the effects of different types of irradiation-induced defects on the flux creep in oxygen-irradiated YBCO coated conductors^27^. To understand the competition between defects in the Au-irradiated film, the influence of Au-, O-irradiation, and annealing on the temperature dependence of the creep rate (*S*) at applied magnetic fields of *μ*_*0*_*H* = 1 T is presented in Fig. S6. The MOD-grown Y(Dy)BCO films show low S values for a broad range of temperatures, which may be due to the strong pinning from pre-existing Y(Dy)_2_O_3_ nanoparticles. The nearly identical *S*(*T*) data on Au- and O- irradiated films shows that the Au- and O- ion irradiation with optimized dosage have the same effects on creep. The ion irradiation introduces a high density of cluster and point defects, which dramatically increases S and reduces the dip depth by reducing the activation energies for vortex depinning. The high density of point defects is responsible for the increasing of *S*(*T*) as a “background” from weak collective pinning. As confirmed by the EELS results, the low temperature oxygen anneal partially removes the point defects, and consequently we can assume an increase in Δ*S* (difference between local maximum and minimum), similar to the changes in the O-irradiated sample.

## Conclusion

In conclusion, this study analyzes the reversible dynamic behavior of oxygen in 2G Y(Dy)BCO coated conductors during irradiation-annealing process. Irradiation of the Y(Dy)BCO wire with 18 meV Au^5+^ ions generates highly oxygen deficient amorphous nanoclusters and randomly distributed point defects. The atomic resolution EELS analysis indicates that the subsequent annealing processes lead to the restoration of oxygen loss in nanoclusters and the removal of plane site oxygen vacancies. Consequently, the depression of *T*_*c*_ and self-field *I*_*c*_ in the irradiated film is partially recovered after annealing. The progressive changes in superconducting property are consistent with the recovery of Y(Dy)BCO lattice by defect annihilation. our findings provide a complete understanding of the irradiation-annealing effects on the enhanced pinning performance of 2G coil wire.

## Methods

### Film fabrication

The samples used in this paper were taken from AMSC’s 2G HTS wire production line. The wire consists of a 75 µm bi-axially textured nickel-tungsten alloy (Ni(5at%)W) substrate fabricated by the Rolling Assisted Biaxially Textured Substrate (RABiTS) technology. A buffer layer stack consisting of a 75 nm Y_2_O_3_ layer, a 75 nm YSZ layer, and a 75 nm CeO_2_ layer was deposited by high-rate reactive sputtering. A 1.2 µm thick YBa_2_Cu_3_O_7-δ_ layer, doped with Dy_2_O_3_ nanoparticles, was deposited by a MOD process. Ag layer with approximately 1 µm was deposited on both sides of the tape.

### Gold irradiation

The samples were irradiated at Brookhaven National Laboratory using the tandem Van de Graaff accelerators. The Au ions with a 5 + charge and an energy of 18 meV were generated from a sputtering source. The samples were irradiated with Au^5+^ ion beam oriented along c-axis of the YBCO. The samples were exposed to the ion beam to achieve a dose of 3 × 10^11^ Au/cm^2^.

### TEM measurements

The cross-sectional and planar-view TEM specimens were prepared by standard mechanical polishing followed by ion milling using Gatan PIPS at 3.2 kV, 4° tilt angle. To prevent the possible reaction, isopropanol was used for sample preparation instead of water. Microstructure and EELS measurements were carried out on FEI Titan 80–300 TEM/STEM with a XFEG source, a monochromator, CEOS hexapole aberration correctors for image and probe lenses, and a high-resolution Gatan Imaging Filter. A collection semi-angle of 55 mrad, dispersion of 0.25 eV/ch and exposure time of 0.1 s/pixel were used for the EELS mapping. Principal component analysis (PCA) was used for the EELS data processing. The EELS Spectrum Image was reconstructed using the first 10 principal components.

## Supplementary information

Supplementary Information.

## References

[1] Foltyn SR (2007). Materials science challenges for high-temperature superconducting wire. Nat. Mater..

[2] Llordés A (2012). Nanoscale strain-induced pair suppression as a vortex-pinning mechanism in high-temperature superconductors. Nat. Mater..

[3] Goyal A (2005). Irradiation-free, columnar defects comprised of self-assembled nanodots and nanorods resulting in strongly enhanced flux-pinning in YBa_2_Cu_3_O_7−δ_ films. Supercond. Sci. Technol..

[4] Wee, S. H., Zuev, Y. L., Cantoni, C. & Goyal, A. Engineering nanocolumnar defect configurations for optimized vortex pinning in high temperature superconducting nanocomposite wires. *Sci. Rep.* **3** (2013). 10.1038/srep02310 PMC3741626 23939231

[5] Cantoni C (2011). Strain-driven oxygen deficiency in self-assembled, nanostructured, composite oxide films. ACS Nano.

[6] Zhang Y (2011). Self-assembled oxide nanopillars in epitaxial BaFe_2_As_2_ thin films for vortex pinning. Appl. Phys. Lett..

[7] van Dover RB (1989). Critical currents near 106 Acm^-2^ at 77 K in neutron-irradiated single-crystal YBa_2_Cu_3_O_7_. Nature.

[8] Civale L (1990). Defect independence of the irreversibility line in proton-irradiated Y-Ba-Cu-O crystals. Phys. Rev. Lett..

[9] Sauerzopf FM (1991). Neutron-irradiation effects on critical current densities in single-crystalline YBa_2_Cu_3_O_7−δ_. Phys. Rev. B.

[10] Civale L (1991). Vortex confinement by columnar defects in YBa_2_Cu_3_O_7_ crystals: Enhanced pinning at high fields and temperatures. Phys. Rev. Lett..

[11] Giapintzakis J (1992). Production and identification of flux-pinning defects by electron irradiation in YBa_2_Cu_3_O_7−x_ single crystals. Phys. Rev. B.

[12] Kwok W-K (2016). Vortices in high-performance high-temperature superconductors. Rep. Prog. Phys..

[13] Sadovskyy IA (2016). Toward superconducting critical current by design. Adv. Mater..

[14] Vlcek BM (1993). Role of point defects and their clusters for flux pinning as determined from irradiation and annealing experiments in YBa_2_Cu_3_O_7-δ_ single crystals. Phys. Rev. B.

[15] Nakashima K (2007). Effect of ion-irradiation and annealing on superconductive property of PLD prepared YBCO tapes. Physica C (Amsterdam, Neth.).

[16] Strickland NM (2017). Effect of annealing high-dose heavy-ion irradiated high-temperature superconductor wires. Nucl. Instrum. Methods Phys. Res. Sect. B.

[17] Ozaki T (2016). A route for a strong increase of critical current in nanostrained iron-based superconductors. Nat. Commun..

[18] Kwon J-H (2018). Extended electronic structure inhomogeneity created by double chain layer defects surrounding columnar tracks in heavy-ion irradiated YBa_2_Cu_3_O_7−δ_. Supercond. Sci. Technol..

[19] Zhu Y, Cai ZX, Budhani RC, Suenaga M, Welch DO (1993). Structures and effects of radiation damage in cuprate superconductors irradiated with several-hundred-MeV heavy ions. Phys. Rev. B.

[20] Yan Y, Kirk MA (1998). Observation and mechanism of local oxygen reordering induced by high-energy heavy-ion (U^+^, Au^+^, Xe^+^) irradiation in the high-*T*_*c*_ superconductor YBa_2_Cu_3_O_7-δ_. Phys. Rev. B.

[21] Kirk MA, Yan Y (1999). Structure and properties of irradiation defects in YBa_2_Cu_3_O_7−x_. Micron.

[22] Rupich MW (2007). The development of second generation HTS wire at American superconductor. IEEE Trans. Appl. Supercond..

[23] Rupich M (2013). Second generation wire development at AMSC. IEEE Trans. Appl. Supercond..

[24] Rupich MW (2016). Engineered pinning landscapes for enhanced 2G coil wire. IEEE Trans. Appl. Supercond..

[25] Xia JA (2007). TEM observation of the microstructure of metal-organic deposited YBa_2_Cu_3_O_7−δ_ with Dy additions. Supercond. Sci. Technol..

[26] Griffith ML, Halloran JW (1992). Pinning in proton-irradiated and annealed YBa_2_Cu_3_O_7-x_ single crystals. Phys. Rev. B.

[27] Eley S (2017). Decoupling and tuning competing effects of different types of defects on flux creep in irradiated YBa_2_Cu_3_O_7−δ_ coated conductors. Supercond. Sci. Technol..

[28] Gauquelin N (2014). Atomic scale real-space mapping of holes in YBa_2_Cu_3_O_6__*+*__δ_. Nat. Commun..

[29] Tolpygo SK, Lin J-Y, Gurvitch M, Hou SY, Phillips JM (1996). Effect of oxygen defects on transport properties and *T*_*c*_ of YBa_2_Cu_3_O_6+x_: Displacement energy for plane and chain oxygen and implications for irradiation-induced resistivity and *T*_*c*_ suppression. Phys. Rev. B.

[30] Biswal R (2008). Point defects creation by swift heavy ion irradiation induced low energy electrons in YBa_2_Cu_3_O_7−y_ through dissociative recombination. AIP Conf. Proc..

