# Supplementary material for: Dynamic behavior of reversible oxygen migration in irradiated-annealed high temperature superconducting wires
Source: Sci Rep. 2020 Sep 10;10:14848. doi: 10.1038/s41598-020-70663-1 (PMC7484753; doi:10.1038/s41598-020-70663-1)
Supplement: Supplementary file 1 — Supplementary Information. [file 41598_2020_70663_MOESM1_ESM.docx]

**Dynamic Behavior of Reversible Oxygen Migration in Irradiated-Annealed High Temperature Superconducting Wires**

Yi Zhang^1^, M. W. Rupich^2^, Vyacheslav Solovyov^3^, Qiang Li^4^ and Amit Goyal^1,*^

^1.^ Research and Education in Energy, Environment & Water (RENEW), State University of New York (SUNY), Buffalo, NY 14260

^2.^ AMSC, 114 E. Main St. Ayer, Massachusetts, 01432

^3.^ Brookhaven Technology Group, 1000 Innovation Rd. Stony Brook, NY 11794

^4.^ Condensed Matter Physics and Materials Sciences Department, Brookhaven National Laboratory, Upton, NY 11973

^*^  Corresponding author: agoyal@buffalo.edu

**Figure captions:**

**Figure S1.** Both (a) irradiated and (b) annealed films have high density of stacking faults, identified as 124 intergrowths, with extra Cu-O chains. The stacking faults are separated by ~1-2 unit cells along c-axis.

**Figure S2.** TEM images of non-irradiated Y(Dy)BCO film show preexisting defects.

**Figure S3.** Two-dimensional EELS map is acquired from non-irradiated film. (a) Two-dimensional spectrum imaging, relative composition maps of Cu-*L_2, 3_*, O-*K*, and Ba-*M_4, 5_* edges. (b) Elements distributions are extracted from the spectrum imaging area. The relative composition profile shows homogeneous distribution of elements.

**Figure S4.** Two-dimensional EELS maps are acquired from (a), (b) irradiated film, and (c), (d) annealed film. The relative composition of irradiated film (b) is extracted from the dashed area in the HAADF image (a). The two-dimensional ADF map and relative composition maps of Cu, O, and Ba are shown in (c). The elements distribution in (d) is extracted from (c) by summing the spectra. The blue area indicates the defect area.

**Figure S5.** In non-irradiated film, the spectra show splitting Cu-*L_3_* edge in chain layer and peak *a* in plane layer.

**Figure S6.** The temperature dependence of the creep rate (*S*) at applied magnetic fields of *μ_0_H* = 1T for as-grown, O-irradiated, Au-irradiated, and annealed samples. The data is extracted from Fig. 6 in reference 27 and Fig. 3 in reference 24 with permission.

**Figure S1.** Both (a) irradiated and (b) annealed films have high density of stacking faults, identified as 124 intergrowths, with extra Cu-O chains. The stacking faults are separated by ~1-2 unit cells along c-axis.


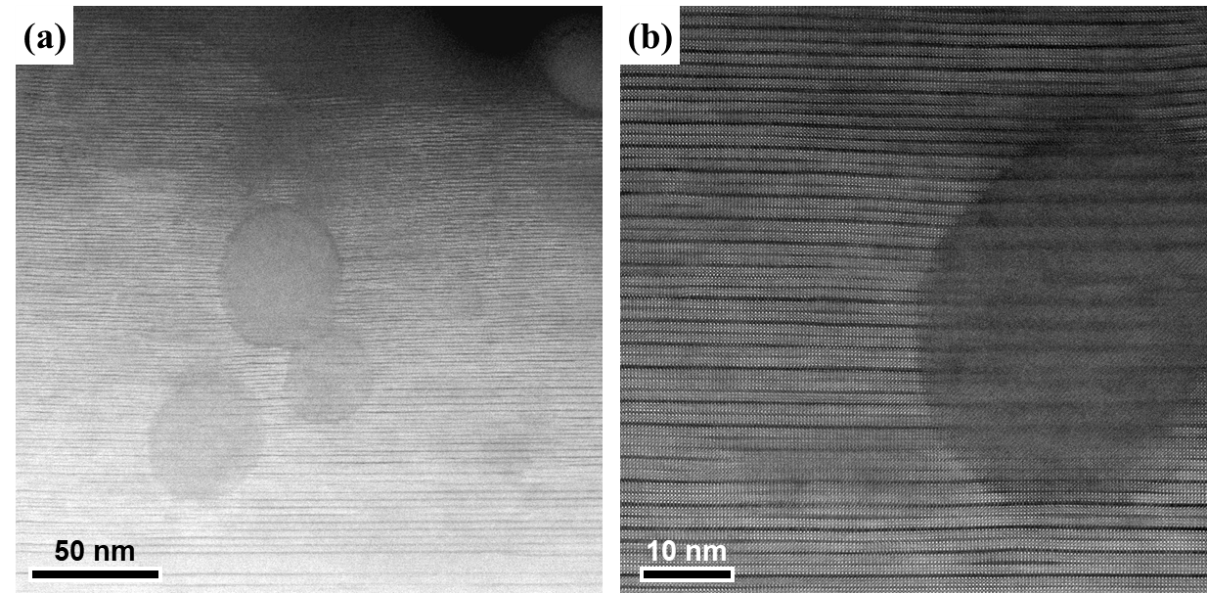


**Figure S2.** TEM images of non-irradiated Y(Dy)BCO film show preexisting defects.


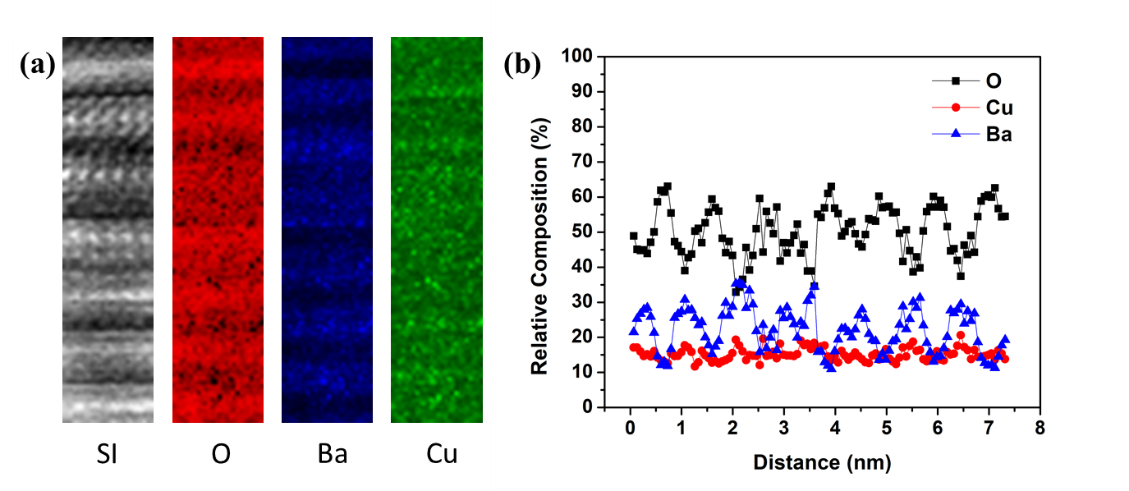


**Figure S3.** Two-dimensional EELS map is acquired from non-irradiated film. (a) Two-dimensional spectrum imaging, relative composition maps of Cu-*L_2, 3_*, O-*K*, and Ba-*M_4, 5_* edges. (b) Elements distributions are extracted from the spectrum imaging area. The relative composition profile shows homogeneous distribution of elements.


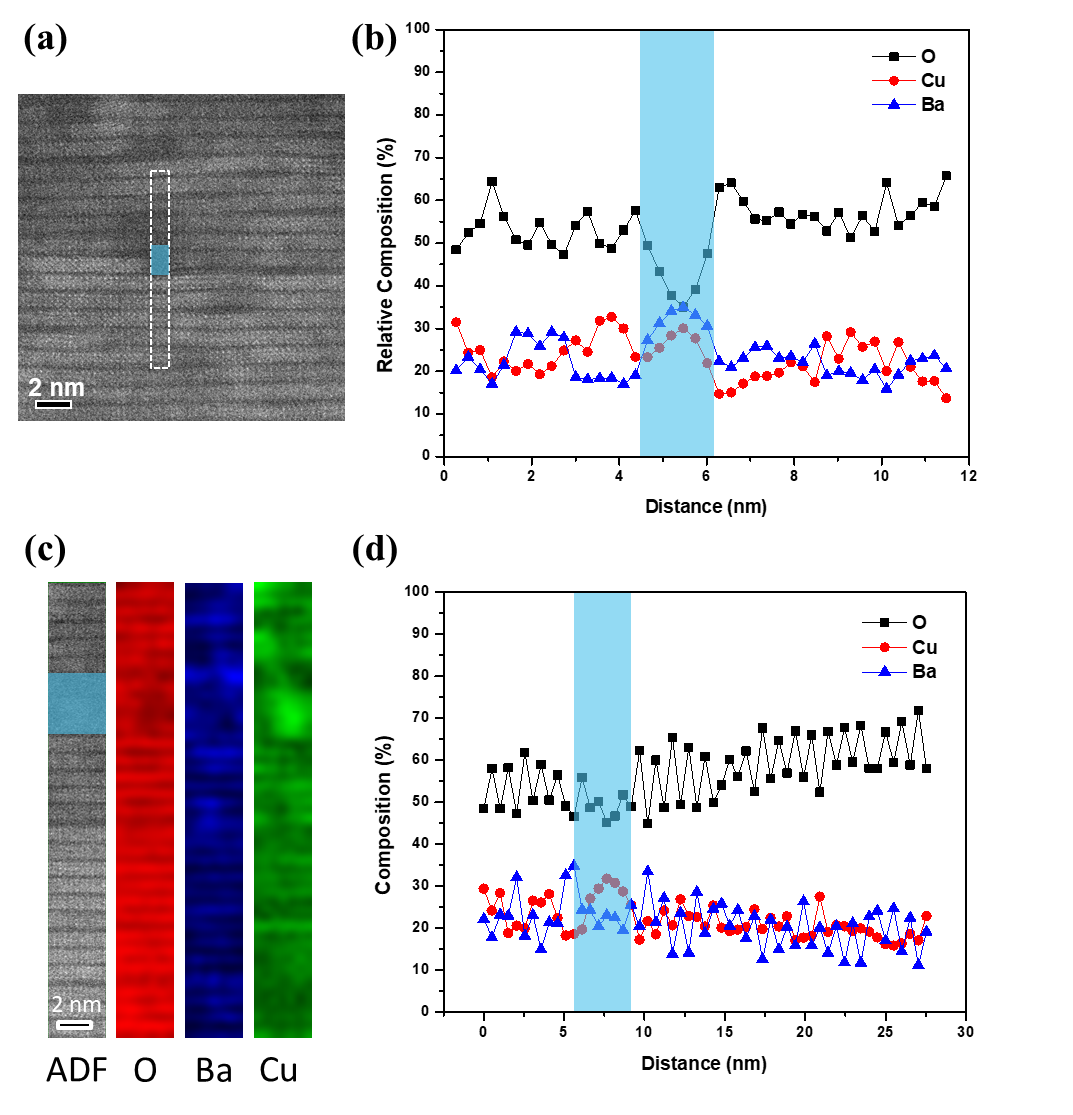


**Figure S4.** Two-dimensional EELS maps are acquired from (a), (b) irradiated film, and (c), (d) annealed film. The relative composition of irradiated film (b) is extracted from the dashed area in the HAADF image (a). The two-dimensional ADF map and relative composition maps of Cu, O, and Ba are shown in (c). The elements distribution in (d) is extracted from (c) by summing the spectra. The blue area indicates the defect area.

**Figure S5.** In non-irradiated film, the spectra show splitting Cu-*L_3_* edge in chain layer and peak *a* in plane layer.

**
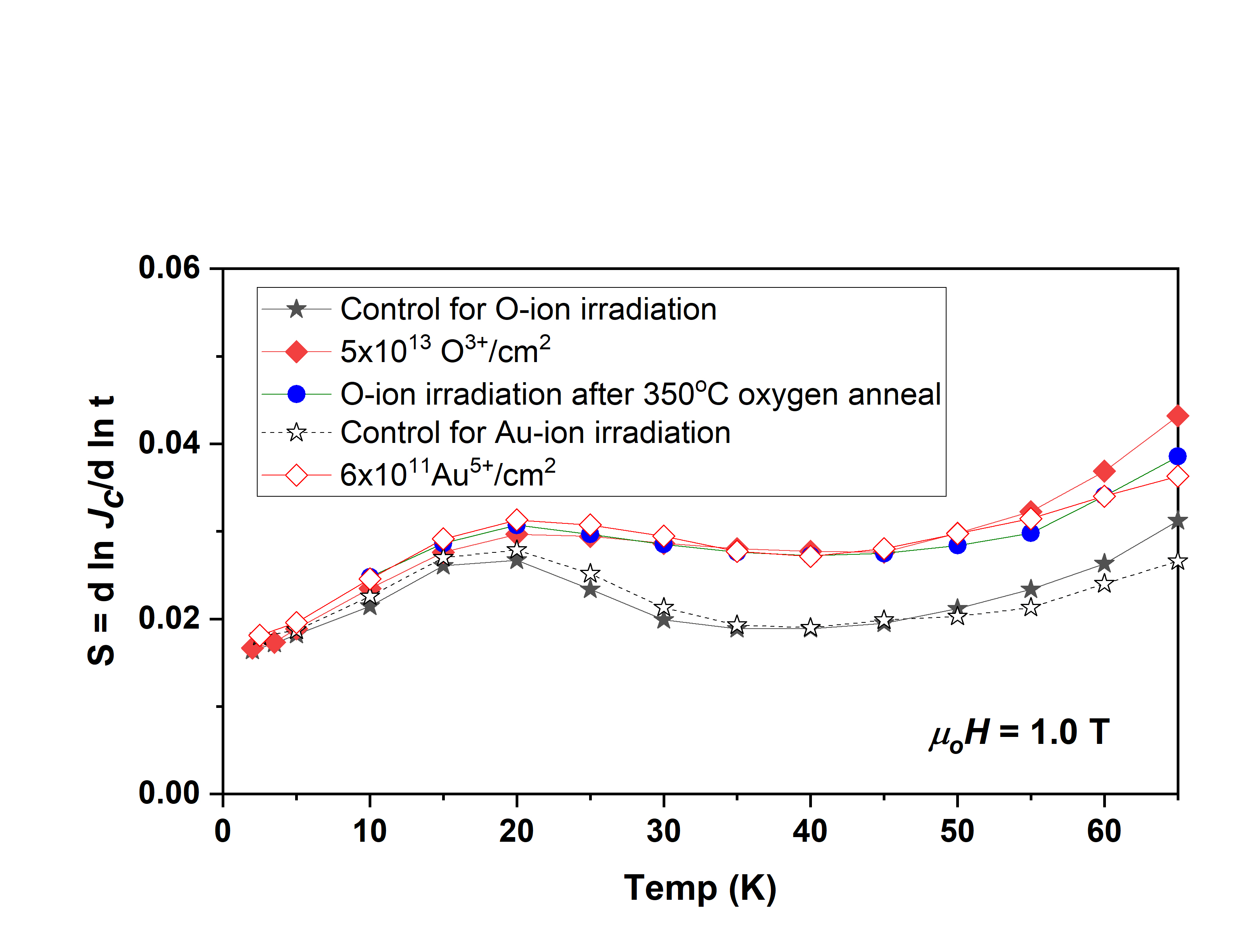
**

**Figure S6.** The temperature dependence of the creep rate (*S*) at applied magnetic fields of *μ_0_H* = 1T for as-grown, O-irradiated, Au-irradiated, and annealed samples. The data is extracted from Fig. 6 in reference 27 and Fig. 3 in reference 24 with permission.
